# Extracellular Vesicles: An Emerging Nanoplatform for Cancer Therapy

**DOI:** 10.3389/fonc.2020.606906

**Published:** 2021-02-08

**Authors:** Yifan Ma, Shiyan Dong, Xuefeng Li, Betty Y. S. Kim, Zhaogang Yang, Wen Jiang

**Affiliations:** ^1^ Department of Chemical and Biomolecular Engineering, The Ohio State University, Columbus, OH, United States; ^2^ Department of Radiation Oncology, University of Texas Southwestern Medical Center, Dallas, TX, United States; ^3^ School of Life Sciences, Jilin University, Changchun, Jilin, China; ^4^ Shenzhen Luohu People’s Hospital, The Third Affiliated Hospital of Shenzhen University, Shenzhen, China; ^5^ Department of Neurosurgery, The University of Texas MD Anderson Cancer Center, Houston, TX, United States

**Keywords:** extracellular vesicle, exosome, therapeutic nanoplatform, cancer therapy, drug delivery

## Abstract

Extracellular vesicles (EVs) are cell-derived membrane particles that represent an endogenous mechanism for cell-to-cell communication. Since discovering that EVs have multiple advantages over currently available delivery platforms, such as their ability to overcome natural barriers, intrinsic cell targeting properties, and circulation stability, the potential use of EVs as therapeutic nanoplatforms for cancer studies has attracted considerable interest. To fully elucidate EVs’ therapeutic function for treating cancer, all current knowledge about cellular uptake and trafficking of EVs will be initially reviewed. In order to further improve EVs as anticancer therapeutics, engineering strategies for cancer therapy have been widely explored in the last decade, along with other cancer therapies. However, therapeutic applications of EVs as drug delivery systems have been limited because of immunological concerns, lack of methods to scale EV production, and efficient drug loading. We will review and discuss recent progress and remaining challenges in developing EVs as a delivery nanoplatform for cancer therapy.

## Introduction

Cell-secreted extracellular vesicles (EVs) have attracted considerable attention over the last decades. These micro- or nano-sized membrane vesicles derived from various cell types are enriched in biological fluids, such as blood plasma, serum, saliva, and urine (1–4). According to disparate biogenesis, EVs can be primarily characterized into three groups: microvesicles (MVs), exosomes, and apoptotic bodies (5). MVs, with a diameter of 100–1,000 nm, are released by outward budding and fission of the plasma membrane. Growing evidence has shown that MVs can package bioactive molecules, nucleic acids (microRNAs (miRNAs) or mRNAs), and proteins that are reflective of the original cell type (6, 7). Similarly, exosomes also contain massive and complex cargos of contents derived from parent cells. Compared with MVs, exosomes are in nanoscale sizes ranging from 30 to 150 nm and mainly originate from intracellular multivesicular bodies; then they are released into the extracellular environment by fusing multivesicular bodies with the cell membrane (8–10). Such distinction suggests that exosomes are more tightly associated with neighbor cell-to-cell communication, whereas MVs may cover distant transportation, thus enabling packaged bioactive effectors to distal sites. Other than MVs and exosomes, apoptotic bodies, with larger diameters of 500 nm to 5 μm, are shed by cells throughout the execution phase of the apoptotic process; their function and related mechanisms are not clearly understood (11). Altogether, EVs that contain diverse cargos (proteins, RNA, DNA, and lipids) trafficked from host cells can be applied not only as mere probes for mechanistic physiology research but also as therapeutic and diagnostic agents for many diseases (12).

EVs have been identified as critical mediators of intercellular communication in both normal and pathological physiological processes, such as cell maintenance and differentiation, tissue regeneration, immune modulation, and tumor development (13–16). EVs are known to be involved in a wide range of processes that underlie tumor development, including tumor microenvironment modulation, angiogenesis, lymphogenesis and tumor invasion, progression, and metastasis (17). EVs that originated from tumor cells have been acknowledged to play a crucial role in driving tumor microenvironment cells toward exacerbating tumor development. These EVs also include specific tumor antigens that can activate a series of immunogenic responses for cancer immunotherapy. Moreover, EVs released from immune cells are known to carry specific proteins and endosome-associated peptides and can induce antigen-specific immune responses for tumor suppression, while EVs derived from mesenchymal stem cells (MSCs) are believed to be paracrine mediators for regulating immune-modulating capacity and tend to accumulate at the tumor site (18, 19). Because of their endogenous functionalities, EVs have been implicated in many aspects of cancer development and, thus, are promising biomarkers and therapeutic candidates for cancer treatment (16, 20–24). Despite multiple advantages, the application of EVs has been limited because of insufficient production and relatively low loading of the desired bioagents. Based on these inadequacies, many approaches have been explored to boost yield and incorporate biomolecules and nucleic acids into EVs for cancer therapy (25–28). Among them, physicochemical technologies, including electroporation, sonication, extrusion, freeze-thaw cycles, membrane permeabilization, and surface functionalization, have been developed to stimulate cells to secrete sufficient amount of EVs, and extracellularly or intracellularly load cargos. Another example of a technology is modification for generating engineered EVs. Besides these exogenous approaches, endogenous methods that highly rely on biological approaches at the cellular level are also known to lead to mass production of EVs and effectively enable bioagent packaging. All these methods make EVs as more attractive delivery vehicles in cancer therapy. When combined with conventional cancer therapy such as chemotherapy and immunotherapy, EVs-based antitumor treatment can be a promising candidate for future clinical application.

## Extracellular Vesicles’ Role in Cancer Development and Its Therapeutic Implications

**Figure 1:**
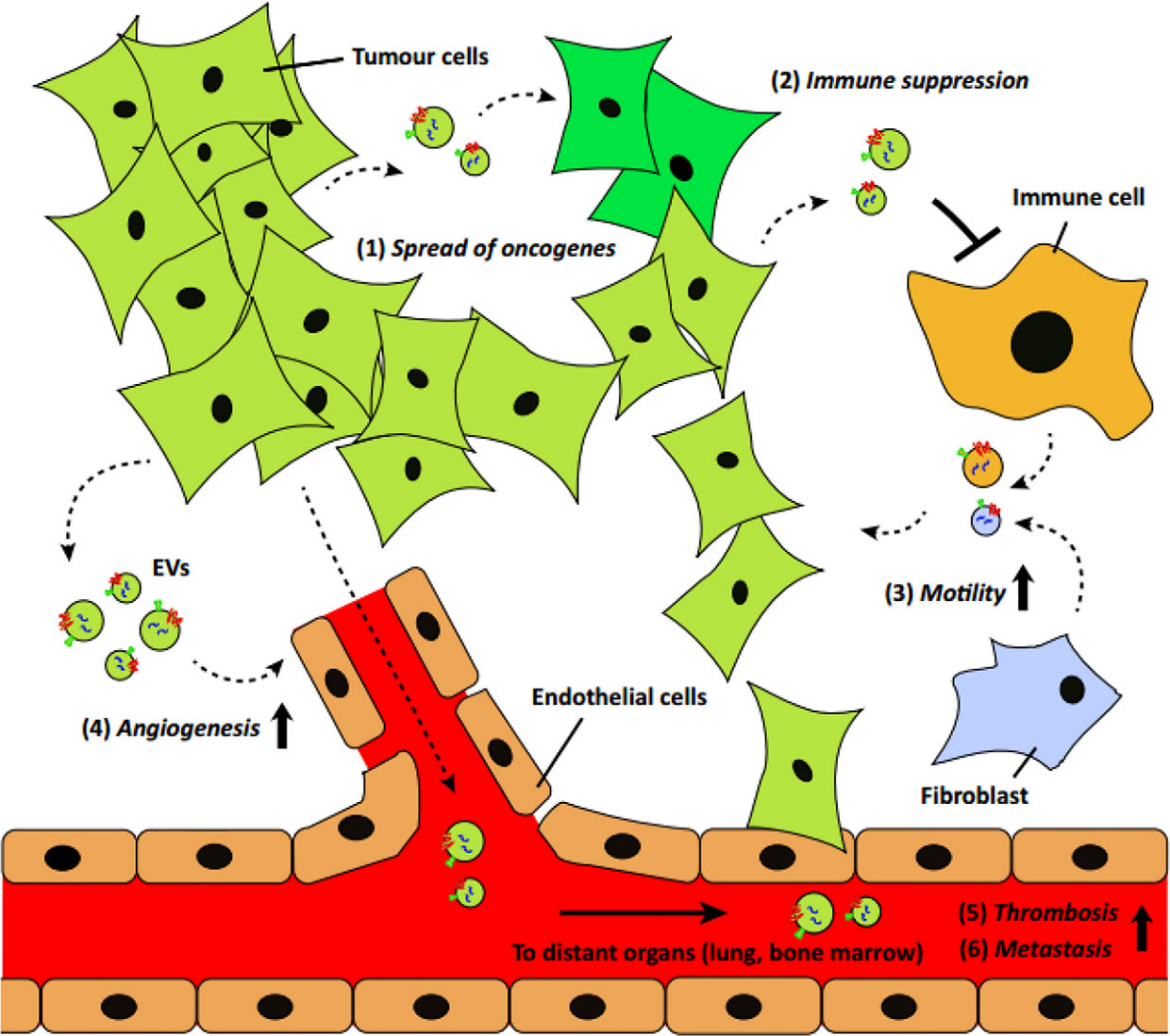
A schematic of EV’s role in cancer development and progression (31) (with reproduction permission).

Readily found in different body fluids, the number of circulating EVs is significantly higher in cancer patients, as reported in previous studies (7, 29, 30). Because of its transmission capacity in the tumor microenvironment or specific distant sites, these EVs have increasingly become recognized as key players in the cellular processes related to cancer pathogenesis, including tumorigenesis, angiogenesis, tumor invasion, progression, and metastasis (**Figure 1**). When it comes to the source of circulating EVs, tumor cells are the first to dominate cancer development and affect other cells for tumor progression. As the initial step in cancer development, tumor formation involves the accumulation of genetic alterations. Tumor-derived EVs (TEVs) have been shown to directly contribute to the spread of phenotypic transformation by transferring their oncogenic traits, and therefore enroll normal cells into the tumorigenic process, enhancing tumorigenesis (32). As a major component of the tumor stroma, fibroblasts can be activated to a cancer-associated phenotype (cancer-associated fibroblasts (CAFs)) in the tumor microenvironment (33). With an abundance of TEVs and increased TGF-β, fibroblasts were shown to undergo transformation from a normal to a CAF-like phenotype when prostate cancer developed; therefore, TEVs appear to facilitate tumorigenesis (34). Another study suggested that apoptotic bodies derived from cancer cells can transfer tumor DNA from H-Ras^V12^-and human c-myc-transfected rat embryonic fibroblasts (REF) to wild-type mouse embryonic fibroblasts (MEF), activating normal fibroblasts and developing the tumorigenic potential of other wild-type cells (35). Additionally, TEVs can support cancer cells to evade apoptosis and promote malignancy in recipient tumor cells. Activating PI3K/AKT and MAP/ERK pathways mediated by TEVs is a common route and has been verified in multiple cancers (36–38). Besides, a protein known as chloride intracellular channel-1 (CLIC1), found to be enriched in some TEVs, can be transferred *via* EVs into other recipient tumor cells and, thus, enhance tumor growth (39).

Tumor growth beyond microscopic size highly relies on increased angiogenesis, a process defined as the new formation of blood vessels for adequate nutrient and oxygen supply throughout tumor development. The communication promoted between tumor cells and endothelial cells and, in particular, with TEVs can facilitate endothelial angiogenic responses for further tumor progression. In most cases, angiogenesis is enhanced by upregulating endogenous vascular endothelial growth factor (VEGF) and other angiogenic signaling pathways in endothelial cells, which are ascribed to the pro-angiogenic effects of tumor-derived EVs. Many studies have reported that a series of proangiogenic cytokines are enriched in tumor-derived EVs, such as VEGF, fibroblast growth factor (FGF), interleukin (IL)-6, IL-1α, and tumor necrosis factor alpha (TNF-α), which directly contribute to tumor angiogenesis (40, 41). Moreover, TEVs have also been shown to promote angiogenesis by transferring genetic information, including messenger RNAs (mRNAs) (e.g., hypoxia inducible factor 1 (HIF-1) and VEGF), long noncoding RNA (lncRNA) (e.g., H19 lncRNA, lncRNA POU3F3 and lncRNA CCAT2), microRNAs (miRNAs) (e.g., miR-9, miR-21, miR-105, miRNA-141-3p, miR-142-3p and miR-210), and chemokine receptors (e.g., EGFR and CXCR4) (42–46). Notably, hypoxic conditions, which are often associated with tumor aggressiveness, can not only merely produce TEVs but also exert a more pronounced effect of these TEVs on ECs than the TEVs released by normoxic tumor cells. According to a glioblastoma multiforme (GBM) model in a previous study, exosomes derived from hypoxic GBM cells can program endothelial cells to secrete potent cytokines, stimulate PI3K/AKT signaling in pericytes, and, thus, facilitate tumor vascularization and pericyte vessel coverage (47). Moreover, hypoxia also results in tumor microenvironment acidification, which could further bolster tumor EVs trafficking within relevant cells and support tumor progression (48).

During tumor progression, several categories of EVs are actively involved in exerting immunostimulatory and immunosuppressive effects on the host’s immune system and counteract antitumor activities. EVs, especially TEVs involved in tumor progression, are known to be closely associated with inflammatory responses and are actively engaged in deregulating the activity of various types of neighboring immune cells. Macrophages are present in all stages of tumor progression; many studies have shown that EVs released by tumor cells can transfer their cargo to macrophages and induce inflammatory responses and promote cancer progression. For example, EVs released by ovarian cancer cells could transfer oncogenic miR-1246 to M2-type tumor-associated macrophages (TAMs) and induce the secretion of tumor-supportive factors, including IL-10 and metalloproteinases (MMPs), while miR-25-3p and miR-921-3p present in EVs secreted by liposarcoma cells could stimulate the secretion of the pro-inflammatory cytokine IL-6 from macrophages, thus triggering tumor progression (49–51). Apart from macrophages, lymphocytes can be significantly modulated by TEVs for tumor progression. Recently, tumor immune evasion regulated through the inhibition of T cell function was found as a result of the binding of programmed death ligand-1 (PD-L1) to programmed cell death protein-1 (PD1). Growing evidence has also showed that PD-L1 highly expressed in TEVs from different cancer types could inhibit T cell functions and contribute to tumor progression (52–54). Furthermore, miRNAs such as miR-24-3p were found to be incorporated within TEVs, triggering T-cell dysfunction and leading to lower patient survival. Admittedly, TEVs play a crucial immunosuppressive role, which can affect the behavior of most immune cells and thus accelerate tumor progression. Notably, EVs sourced from various cancers were also shown to express specific tumor antigens that could be taken up by immune cells to induce profound tumor-specific immunological enhancement responses. From this viewpoint, TEVs that are generally considered as promoters of tumor progression can also work as therapeutic agents or cancer diagnosis and treatment signals. For instance, EVs shed by melanoma cell lines were reported to effectively activate macrophages and improve the maturation of dendritic cells (DCs) and T-cell proliferation, which subsequently promoted antitumor effects (55). In other studies, DCs primed with EVs originated from mesothelioma cells and glioblastoma cells could induce potent antitumor responses and significantly increase survival rate (56, 57). These evidence implicated that EVs involved in cancer development could be a promising therapeutic nanoplatforms for cancer treatment.

## Engineered Extracellular Vesicles for Cancer Therapy

**Figure 2:**
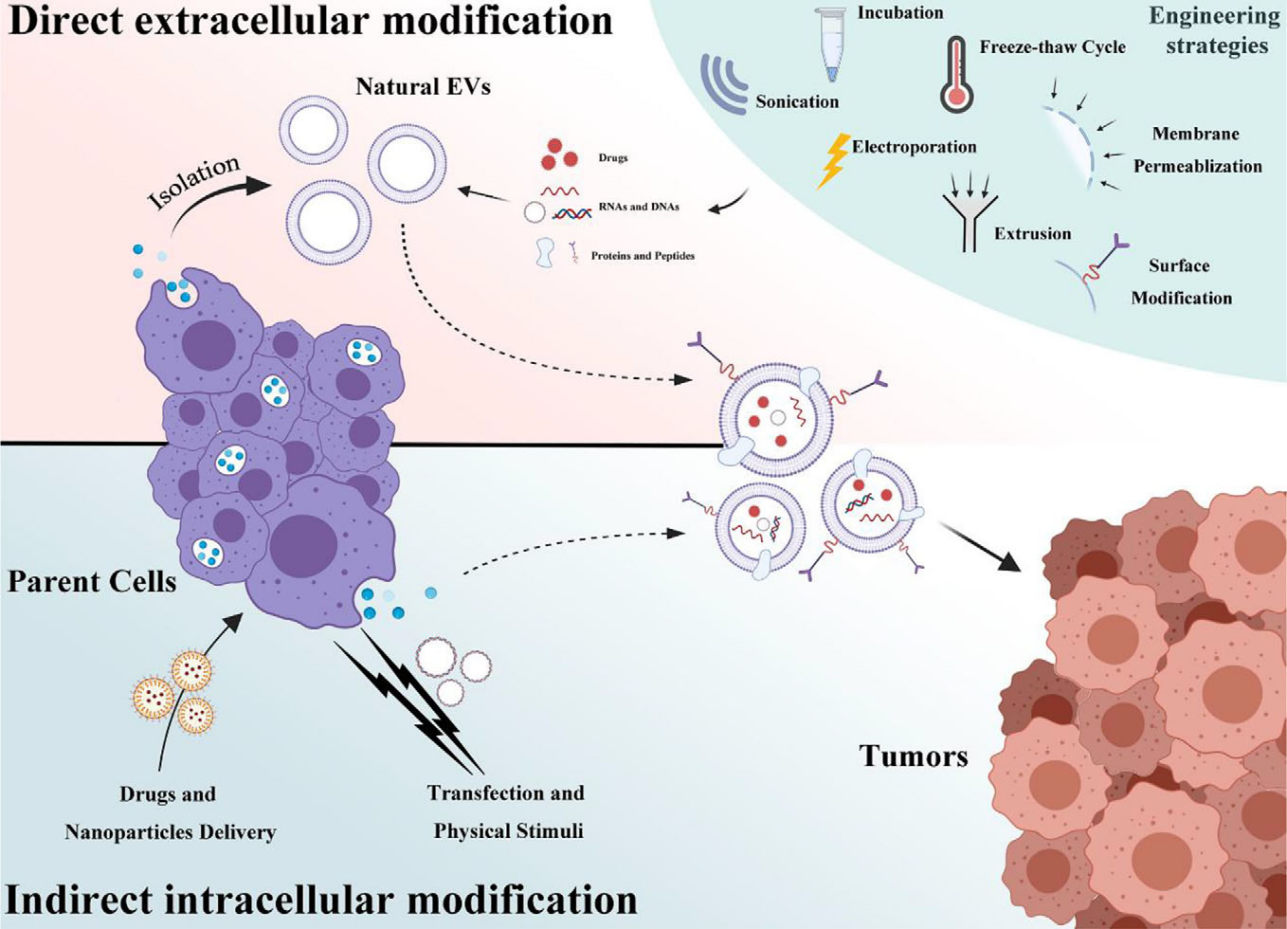
A schematic of EVs engineering strategies that is categorized into two groups: direct extracellular modification and indirect intracellular modification.

EVs have emerged as novel and promising therapeutic agents because they can be used for a wide range of tumor-related processes and deliver therapeutic cargos, including nucleic acids and proteins, into tumor sites. Despite multiple advantages, the application of EVs for anticancer therapy has been limited because of insufficient production and relatively low loading of the desired bioagents. As such, many approaches have been explored for mass production and for incorporating a substantial amount of nucleic acids and small biomolecules into EVs. These engineering approaches can be primarily categorized into two groups: direct extracellular modification and indirect engineering of donor cells. These engineering strategies involved in current EVs-based cancer therapy will be summarized and discussed (**Figure 2**).

### Direct Extracellular Modification of Extracellular Vesicles for Cancer Therapy

Extracellular modification of EVs often refers to the direct load of cargos such as genetic elements and biomolecules or drugs and surface modification after EV isolation and purification.

#### Post-Load of Cargos into Extracellular Vesicles for Cancer Therapy

Direct encapsulation of cargos into EVs often relies on a series of physical procedures or technologies including incubation, freeze-thaw cycles, electroporation, sonication, extrusion, and membrane permeabilization. Among them, incubation and freeze-thaw cycles are two of the easiest methods for engineering EVs. As for incubation, highly concentrated cargos tend to interact with the vesicle’s lipid biolayers and be passively diffused into the EV cavity (58). Because of its maneuverability, researchers have widely used incubation to modify EVs for treating multiple diseases (59–61). When it comes to cancer treatment, small nucleic acids and chemotherapeutic agents (paclitaxel (PTX) and doxorubicin (DOX)) are common cargos co-incubated with endogenous EVs. In a study by Saari et al., PTX was incubated with extracted exosomes derived from prostate cancer cells at 22 °C, and the resulting loaded vesicles could enhance PTX’s cytotoxic effect when treating autologous prostate cancer (62). However, the low loading capacity as well as the limited selectivity of encapsulated cargos are considered main challenges for applying this method. Alternatively, the freeze-thaw cycle is also a straightforward method where the desired cargos are incubated with EVs combined with RT, and then the mixture is processed with repeated cycles from freezing in −80 °C or liquid nitrogen to RT re-thawing to encapsulate cargos. Following this method, Goh et al. developed DOX-loaded EVs (derived from monocytes) to preferentially target cancer cells and induce apoptosis (63). Like the incubation method, the freeze-thaw cycles method for EVs engineering also suffers from inefficient cargos loading, which can be ascribed to passive diffusion as well as severe aggregation during the cycles.

**Figure 3:**
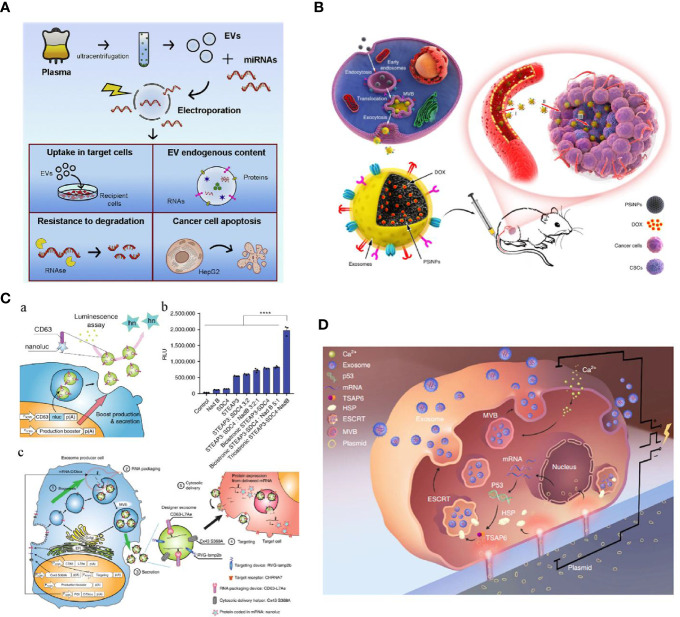
**(A)** Optimized electroporation parameters for direct EVs loading were screened including voltages and pulses. When the desired cargos were miRNAs, 750 V and 10 pulses showed the most significant miRNA enrichment (66). **(B)** A novel tumor-cell-exocytosed exosome-biomimetic porous silicon nanoparticles (PSiNPs) was developed through the incubation of the synthetic nanoparticles with parent cells. Such engineered exosomes significantly increased DOX loading and improved antitumor efficacy (67). **(C)** An exosomal transfer into cells (EXOtic) device enables exosome mass production and efficient exosomal mRNA delivery (68). **(D)** A cellular nanoporation (CNP) technology trigger a 50-fold increase of exosomes yield and thousand-fold increase of mRNA transcripts in the released exosomes from CNP-transfected cells release (69) (with reproduction permission).

For efficient cargo loading, many physical strategies have been introduced to optimize EV engineering in cancer therapy. As an established non-viral technique for traditional extracellular delivery, electroporation has been extensively used for direct engineering of EVs (64). Similarly to cell structure, EVs are composed of a bilayer lipid membrane, which allows genetic elements, small molecules, or drugs to be incorporated into their structure through pores induced by the electric field. Generally, the loading efficiency can be affected by voltage, pulse times, pulse interval, and protection buffer, and highly depends on the category of loading cargos. Cargo encapsulation into EVs *via* conventional electroporation is generally low because the lipid membrane structure of the EVs can be damaged under electric fields. Therefore, many electroporation protocols have been developed to optimize this procedure and efficiently engineer EVs. Zhang et al. introduced an miRNA loading strategy in virtue of calcium chloride-mediated electroporation into EVs for *in vitro* and *in vivo* delivery. Their results showed that specific miRNAs in exosomes could be efficiently manipulated *via* modified transfection with buffer protection (65). Nonetheless, such transfection may be associated with contaminating transfection reagents (buffer or residue cargos) in the resulting engineered EVs products, possibly impairing subsequent applications. As shown in **Figure 3A**, Pamotto et al. screened different electroporation parameters including voltages and pulses, and found that setting with 750 V and 10 pulses allowed significant miRNA enrichment. Two kinds of engineered EVs that incorporated antitumor miRNAs (miR-31 and miR-451a) showed they could silence target genes involved in anti-apoptotic pathways and, thus, promote apoptosis of the HepG2 hepatocellular carcinoma cell lines (66). In another study associated with engineered EVs for treating pancreatic cancer, Kamerkar et al. also used electroporation to enrich specific siRNA or shRNA in EVs for targeting KRAS^G12D^, a common mutation in the GTPase KRAS involved in pancreatic ductal adenocarcinoma (PDAC). With a specific electroporation buffer and Gene Pulser Xcell Electroporation System (parameters: 400 V, 125 μF and ∞ ohms), KRAS^G12D^ siRNA and shRNA plasmid could be efficiently encapsulated into isolated and purified exosomes. These KRAS^G12D^-related engineered exosomes (also termed as iExosomes), together with the modification of CD47 peptides on the surface of iExosomes for escaping phagocytosis, can significantly suppress tumor growth in multiple mouse models of pancreatic cancer and support increased survival (70). On the basis of these promising preclinical results, a clinical trial using iExosomes to target metastatic pancreatic cancer was recently registered (NCT03608631). Together with other specific RNAs enriched in EVs for treating multiple diseases (i.e., miR-124 enriched MSCs’ EVs for acute ischemic stroke, NCT03384433), these clinical trials will hopefully give us further insight into the potential of EVs as drug delivery vehicles for RNA species.

Other than electroporation, alternative approaches such as sonication, extrusion, and membrane permeabilization have also been used to engineer EVs with therapeutic agents for cancer therapy. Sonication has emerged as a potential strategy for generating engineered EVs. When compared to electroporation, EVs engineering through sonication can prevent charged cargo aggregation and also distribute cargos for cancer treatment in the EVs. Lamichhane et al. reported the efficient application of sonication for actively delivering oncogenic siRNAs into EVs. In their study, EVs could be loaded with small RNAs with no aggregation; these therapeutic EVs were taken up by HEK293T and MCF-7 and could target HER2 knockdown, thus resulting in tumor growth inhibition (71). In addition to the nucleic acids, small molecular drugs such as chemotherapeutic agents have also been applied to engineered EVs for cancer therapy. After isolating and purifying exosomes from macrophages, Kim et al. applied mild sonication to encapsulate PTX for treating multiple drug resistance cancer. According to the results, sonication delivery of PTX showed significantly better loading efficiency than routine RT incubation and electroporation. With the high payload of PTX in the engineered EVs, a potent anticancer effect was further confirmed in a tumor-bearing model (72). Extrusion is another direct extracellular approach to modify EVs. During extrusion with specific porous membrane, the EV lipid membrane tends to be disrupted, following cargzo loading into EVs. Using the extrusion method, Fuhrmann et al. showed that porphyrins could readily be loaded into exosomes collected from diverse stem cells. The authors showed that artificial exosomes produced by the extrusion method had a better anticancer effect than the exosomes prepared *via* traditional passive methods (incubation and freeze-thaw cycles). As a method based on membrane permeabilization, saponin-assisted modification has also been extensively applied to engineering EVs for cancer therapy. Unlike the strategies discussed earlier, saponin-associated approaches are based on surfactant molecules that can generate pores in the EV membrane by interacting with cholesterol during co-incubation.

The engineering methods mentioned earlier allow EVs to be more attractive delivery vehicles for cancer treatment. Physical technology-assisted approaches are definitely more potent and promising than conventional incubation or freeze-thaw cycles, where encapsulation efficiency and loading capacity are often low. Among them, electroporation was shown to be effective for post-loading cargos, while sonication, extrusion, and membrane permeabilization are rarely used. These direct engineering strategies face similar challenges associated with low EV recovery and limited cargo loading efficiency, which impair subsequent application in cancer therapy. With the physical technology applied, membrane disruption cannot be avoided and can only be partially recovered. Moreover, loading capacity and efficiency highly depend on the physicochemical properties of cargos categories and operating loading techniques. As a result, advanced strategies for improving direct EVs modification need to be developed for efficient delivery of therapeutic agents in cancer treatment.

#### Surface Functionalization of Extracellular Vesicles for Tumor Targeting

EV surface is known to include membrane proteins that can contribute to their biodistribution and targeting capabilities. For cancer therapy, interest has grown in surface membrane protein modification to improve EVs’ targeting efficiency to tumor sites for therapeutic functions. Multiple studies have developed extracellular strategies where isolated and purified EVs were post-handled to modify the EV surface. Multivalent electrostatic interactions and receptor-ligand binding are frequently leveraged for surface engineering of EVs. Nakase et al. exploited electrostatic interactions to bind cationic lipids to the surface of exosomes to enhance intracellular release efficiency of encapsulated cargos in the isolated exosomes. Such interactions produced exosomes with a pH-sensitive fusogenic peptide that could promote binding and uptake by targeted HeLa cells, subsequently inducing efficient cytotoxicity (73). In another study by Qi et al., receptor-ligand binding was developed to generate a dual-functional blood exosome-based superparamagnetic vehicle for cancer therapy. By conjugating transferrin-superparamagnetic nanoparticles with blood derived exosomes, enhanced tumor targeting could be observed under an external magnetic field, followed by significantly inhibiting tumor growth (74).

Many covalent approaches were also used to provide more stable surface engineering of EVs. Among multiple covalent strategies, moderate and specific cross-linking reactions are preferred because the reaction conditions for EV surface modification are in place to prevent EVs from potential disruption or aggregation due to additional buffer solution, osmotic stress, and reaction temperature. As a highly specific click reaction, azide-alkyne cycloaddition can work in aqueous media and low temperatures, rendering it ideal for EVs surface modification. Wang et al. developed a combination of metabolic labeling of newly synthesized proteins or glycan/glycoproteins of parent cells with chemically active azide groups and bioorthogonal click conjugation to engineer exosomes (75). After metabolically engineering parent cells, azide-integrated exosomes were generated, and a variety of small molecules and proteins attached on dibenzocyclooctyne-amine were specifically conjugated. These engineered exosomes have showed promise in the biomedical field because they can efficiently deliver to target sites. In a similar study, Smyth et al. developed engineered exosomes with active terminal alkyne groups (76). According to these methods, the amine groups of the exosomal proteins were first cross-linked with the carboxyl group of 4-pentynoic acid using carbodiimide activation. Then, alkyne-functionalized EVs were conjugated with azide-fluorescence groups through a specific click reaction. These covalent approaches for direct EV surface modification seem to have more advantages than the non-covalent ones because click chemistry is considered a non-invasive strategy that allows exogenous molecules to be incorporated onto the EV surface, with no negative effects on EVs’ physicochemical characteristics. However, only a few reports on engineered EVs used for cancer therapy are available. As such, more systematic studies are needed to support their use in cancer treatment, especially targeting efficiency and safety.

### Indirect Intracellular Modification for Engineering Extracellular Vesicles

Indirect intracellular modification is another promising strategy for massively producing artificial EVs. With this approach, parental cells are initially manipulated through physical or genetic approaches, so they can release the engineered EVs. Many cancer researchers have used this concept to endogenously package specific substances into EVs by simply incubating donor cells with the desired cargos, particularly chemotherapeutic agents. In a study conducted by Pascucci et al., PTX was initially co-incubated with bone marrow stromal cells for 24 h. PTX-loaded-exosomes primed with high dose of PTX were then recovered. The results showed that such PTX-engineered exosomes could significantly inhibit the proliferation of CFPAC-1 human pancreatic cells (77). Moreover, studies found that anticancer drugs could stimulate donor cells to secret more EVs with enriched therapeutic agents being packaged, thereby contributing to tumor suppression. According to Lv et al., co-incubating PTX and hepatocellular carcinoma cells (HepG2) *in vitro* could simultaneously increase exosome secretion and exosomal heat shock proteins (HSPs) loading. Notably, these engineered exosomes led to immunogenic responses and, thus, to increased NK cell cytotoxicity toward cancer cells (78). Considering the low efficiency of drug encapsulation, another study (**Figure 3B**) led by Yang et al. developed tumor-cell-exocytosed exosome-biomimetic porous silicon nanoparticles (PSiNPs) as drug carriers, greatly increasing drug loading. The authors claimed that engineered exosomes could be exocytosed from tumor cells after incubating with DOX@PSiNPs. These exosomes effectively augmented *in vivo* DOX enrichment in total tumor cells and significantly improved anticancer efficacy (67). Other than routine chemotherapeutic drugs, other therapeutic substances were also applied for indirect intracellular modification. In a study by Behzadi et al., an HSP70-enriched cell lysate was introduced into J774 cells to obtain the desired exosomes. After incubation, HSP70-loaded exosomes could be recovered from conditioned medium and were found to play an immunoadjuvant role in cancer immunotherapy and significantly delay the occurrence of fibrosarcoma tumors (79). Certainly, not all substances can readily enter parent cells and be encapsulated into EVs by simple incubation because hydrophobic/hydrophilic and charging properties of the substances will greatly influence this process and ultimate loading efficiency. That is, other external assistance and physical or genetic technologies need to be introduced to optimize indirect EV engineering for cancer therapy.

External stresses, such as starvation, hypoxia, and heat treatment through which cells can be activated to produce functional EVs have been gaining interest. For example, previous studies have observed that umbilical-derived MSCs and human leukemia cell line (K562) under hypoxia profoundly elevated EV secretion; these EVs promoted angiogenesis more effectively than EVs from normoxic cells. Further scrutiny revealed that hypoxic exosomes contained abundant amounts of miRNAs (e.g. miRNA-150 and miRNA-210) which could target angiogenesis signaling pathways (80–82). Similar results were reported when cells are under other cellular stresses. Despite the useful strategies used to produce EVs by incorporating a few genetic messages, only moderate EVs can be released and limited cargos can be encapsulated for cancer treatment. To improve modification efficiency, parent cell transfection is the most widely used and efficient method. Ding et al. delivered exogenous miRNA into exosomes produced by human umbilical cord mesenchymal stromal cells (hucMSCs) through lipofectamine transfection. After hucMSCs transfection, miR-145−5p accumulation was detected in hucMSC-derived exosomes, which were shown to effectively inhibit PDAC cells proliferation and invasion *in vitro*, and significantly reduce the growth of xenograft PDAC tumors *in vivo* (83). With the assistance of vector transfection, Ohno et al. also showed that the Let-7a-loaded exosomes could effectively target recipient tumor cells after engineering their surface with targeting peptides (GE11 and EGF). They also showed that intravenously-injected engineered exosomes could accumulate in the tumor site and effectively suppress tumor growth (84). Many studies have recently focused on approaches that cannot merely enrich EVs’ contents but increase EVs’ production. In a recent study by Ibrahim et al., skin fibroblasts engineered by canonical Wnt signaling (overexpress β-catenin and the transcription factor Gata4) were turned to a exosome factory that promoted exosome secretion with specific therapeutic functions (85). Moreover, Ryosuke et al. (**Figure 3C**) developed a novel exosomal transfer into cells (EXOtic) device that enables exosome mass production and efficient exosomal mRNA delivery. By transfecting CD63 plasmids in parent cells with encoding candidates, including STEAP3, syndecan-4 (SDC4), and a fragment of L-aspartate oxidase (NadB), the exosomes yield could be boosted by over 40-fold. Also, a specific packaging device, archaeal ribosomal protein L7Ae, and a cytosolic delivery helper, connexin 43 (Cx43), were used to deliver mRNA into the cytosol of target cells and, thus, produce mRNA-engineered exosomes. By combining these two synthetic bioinspired devices, many exosomes with enriched cargos could be delivered to the desired sites for therapeutic functions (68). Because of the exciting results found for treating other diseases, these promising biological endogenous EVs engineering methods can provide a new horizon of engineered EVs for cancer therapy. In addition to endogenous loading *via* lipid transfection and biological approaches, physical transfection is another promising strategy for therapeutic cargo encapsulation during EV biogenesis. As shown in **Figure 3D**, Yang et al. developed a cellular nanoporation (CNP) technology through which functional mRNAs can be encapsulated into exosomes *via* physical nanoelectroporation. Interestingly, a 50-fold increase of exosomes yield and thousand-fold increase of mRNA transcripts could be observed when compared with traditional bulk electroporation. These PTEN-engineered exosomes were simultaneously functionalized with targeting peptide-modified CD47 and were shown to successfully cross the brain blood barrier for targeting restored tumor suppressor function, enhance inhibition of tumor growth, and increase survival (69). Although these indirect intracellular modification methods can be mass produced and widely enrich EV function, the major challenges are that the amount of active substances encapsulated in EVs cannot be quantified precisely, and the extraction yield and purity of the cargo-loaded EVs obtained by this method are relatively low. Hence, more systematic studies and appropriate analytic tools associated with EVs engineering, and also advanced purification technologies are needed to be developed for applying these therapeutic EVs’ in cancer therapy.

## Extracellular Vesicles’ role in Different Cancer Therapies

Three subsections of EVs-based anticancer treatment are included: cancer chemotherapy, immunotherapy and phototherapy (67, 86–89). Because of their low toxicity and ability to get through biological barriers to reach remote tissues, EV-based platforms have been designed for shuttling chemotherapeutics to the desired tumor tissue. Besides, endogenous functionalities ascribed to self-carrying specific proteins, trans-membrane markers, and tumor antigens make EVs promising immunotherapeutic platforms.

### Extracellular Vesicles Involved in Cancer Chemotherapy

**Figure 4:**
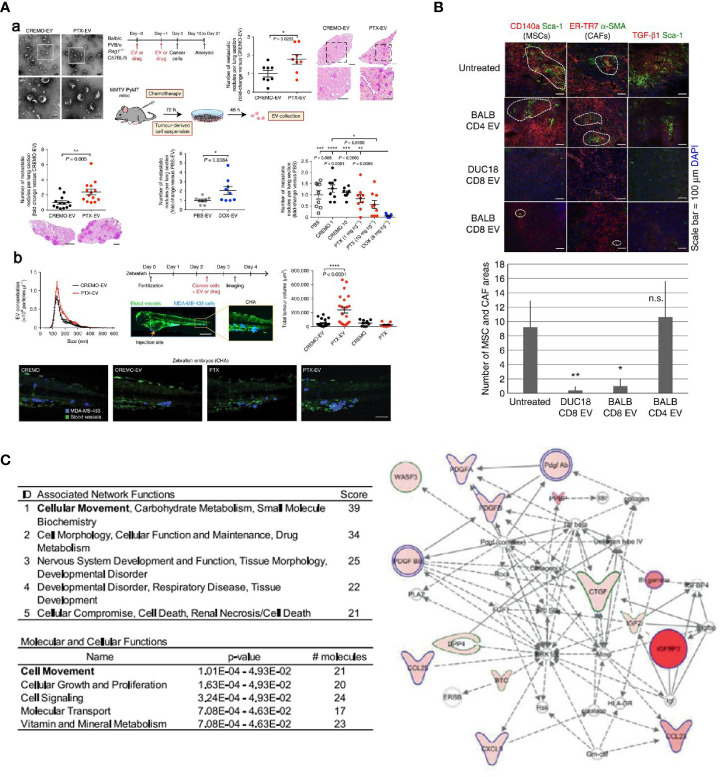
**(A)** EVs elicited from two cytotoxic drugs, taxanes and anthracyclines, were found to enhance the pro-metastatic ability in mouse **(a)** and zebrafish **(b)** tumor models (90). **(B)** EVs collected from activated CD8+ T cells were found to be actively involved in interrupting fibroblastic stroma-mediated tumor progression by depleting tumor mesenchymal cells (91). **(C)** Molecular profile of radiation-derived exosomes demonstrated that radiation increased the exosomal proteins related to tumor migration and progression (92) (with reproduction permission).

Chemotherapy is one of the gold standards in cancer treatment. In addition to cytotoxic side effects, pro-metastatic effects of chemotherapy are important and should be addressed. Keklikoglou et al. reported pro-metastatic effects triggered by chemotherapy in a breast cancer model (90). Two broadly used cytotoxic drugs, taxanes and anthracyclines, were found to enhance the pro-metastatic ability of tumor-derived EVs, which were enriched with annexin A6 (ANXA6), a protein that promotes the activation of NF-κB-dependent endothelial cells (**Figure 4A**). Moreover, ANXA6 was also abundant in EVs of breast cancer patients who received neoadjuvant chemotherapy. The expression of cytokines (e.g., C-C motif chemokine ligand 2 (CCL2)) that facilitate mammary tumor metastasis was also examined using tumor-free and 4T1-mCh tumor-bearing *Rag1* ^−/−^ mice. CCL2 was upregulated in mice treated with PTX. In addition, Ly6C^+^CCR2^+^ monocytes that trigger metastasis of mammary tumors were also found to be higher, which is inconsistent with the expression of CCL2, which is known to enhance the establishment of Ly6C^+^CCR2^+^ monocyte-assisted lung metastasis (93, 94). Also, Andrade et al. found that more EVs were secreted by melanoma cells after temozolomide and cisplatin treatment; these EVs facilitated re-growth of melanoma *in vivo* (95).

Growing evidence indicates that chemotherapy-induced EVs and the cargos they carry caused chemoresistance in addition to altering tumor behavior and promoting inflammation in the tumor microenvironment. Kreger et al. found that EVs secreted from breast cancer MDA-MB231 cells treated with paclitaxel contained abundant surviving cargo molecules associated with therapy resistance (96). In another study, apoptotic glioblastoma multiforme cells after chemotherapeutic treatment could generate more apoptotic EVs enriched with spliceosomes (e.g., RBM111, a representative splicing factor), which would also promote proliferation and therapy resistance *via* altering RNA splicing in recipient cells (97). The same effect was found not only in solid tumors but also in blood cancers. A study conducted using bone marrow stromal cell-derived exosomes also proved to induce drug resistance to bortezomib in myeloma cells (98). Kim et al. investigated chemodrug resistance in multidrug resistance tumor cells using PTX-loaded exosomes (72). According to the results, cytotoxicity was found to increase more than 50 times in drug-resistant tPgp protein-overexpressing Madin-Darby canine kidney MDCK_MDR1_ cells. Moreover, potent antitumor effects were observed when these PTX-loaded exosomes were used in a Lewis Lung Carcinoma (LLC) model through airway delivery. Notably, 97.9 ± 2.0% of exosomes were found to be co-localized with lung metastases, revealing an effective targeting of the exosomes. Similarly, autologous prostate cancer-derived EVs were proved to be effective drug carriers to deliver PTX to cells they originated from and increased cytotoxicity was observed because drugs were delivered through an endocytic pathway (62). In PC-3 cells, the cytotoxic effects could reach the most significant when doses of PTX was over 50 nM, while the cytotoxic effects of PTX at 10 nM and below was significantly weaker. The maximum decrease of PC-3 cells’ viability was around 60% after 48 h, while in LNCaP cells, the maximum decrease of cell viability was up to 80% regardless of the dose. Even though improved cytotoxicity was found when using cancer-cell derived EVs, cancer cell viability was enhanced when non-drug loaded EVs were involved. Therefore, several clinical trials (i.e., NCT01854866) using tumor-derived EVs plus with chemotherapeutic drugs was initiated to further evaluate their performance.

MSC-derived exosomes are good candidates for drug delivery in tumor therapy because of their reduced intrinsic immunogenicity. Melzer et al. incubated MSCs with taxol for 24 h and showed the efficiency of taxol-loaded MSC-derived exosomes in targeting primary tumors and metastases (99). In vitro studies showed that equivalent cytotoxic effects were achieved between exosomes and taxol substances, although a 7.6-fold reduced taxol concentration in exosomes was applied. Also, a 60% reduction of primary tumors and 50% reduction of distant organ metastases were observed in breast tumor mouse models using MSC taxol exosomes and taxol. In contrast, the concentration of taxol in exosomes was 1,000-fold lower than with taxol substances to obtain similar results. Other than stems cells, Tian et al. showed that exosomes derived from mouse immature dendritic cells (imDCs) and encapsulated with doxorubicin (Dox) *via* electroporation with up to 20% efficiency facilitated *in vitro* and *in vivo* anti-tumor effects (87). Briefly, imDCs were engineered to express a well-characterized exosomal membrane protein (Lamp2b) fused to an αv integrin-specific iRGD peptide. Consequently, iRGD exosomes could target αv integrin-positive breast cancer cells. Overall, high targeting efficiency and the ability to inhibit tumor growth without overt toxicity were achieved using this proposed platform. Exosome-biomimetic nanoparticles also exerted satisfactory effects for targeted chemotherapy as drug carriers. As aforementioned (**Figure 3C**), a novel tumor-cell-exocytosed exosome-biomimetic PSiNPs that contained with exosome-sheathed DOX loading was developed for cancer chemotherapy (67). PSiNPs were produced using the electrochemical etching method (100) by stirring the DOX solution with PSiNPs to obtain DOX@E-PSiNPs. Subsequently, cells were treated with either PSiNPs or DOX@PSiNPs to form DOX@E-PSiNPs. This technology resulted in anticancer activity and cancer stem cell reduction in various tumor models with high cellular uptake and cytotoxicity effects.

### Extracellular Vesicle-Based Cancer Immunotherapy

EVs are known to play integral roles in tumor development and progression. According to multiple studies, EVs derived from immune cells are primed with specific proteins and endosome-associated peptides, while EVs secreted by tumor cells contain specific tumor antigens. Because of their endogenous functionalities, EVs have been identified as promising immunotherapeutic nanoplatforms for cancer treatment. In terms of excreting cells, EV-based cancer immunotherapy can be mainly categorized into two groups: TEVs and immune cells-derived EVs (ICEVs).

TEVs are more productive than other kinds of EVs because tumor cells tend to be more aggressive under specific tumor microenvironments and stimuli during development, including pH and hypoxic conditions. As mentioned earlier, these TEVs have been known to play a crucial role in promoting tumor progression by transferring their specific cargos between tumor cells and related cells. Otherwise, these secretions were also found to express specific tumor antigens that could be taken up by immune cells to induce profound tumor-specific CTL responses. Many studies have exploited this TEV function to develop specific immunogenic responses for cancer immunotherapy. In studies by Mahaweni et al. and Liu et al., DCs primed with exosomes that were isolated from mesothelioma cells and glioblastoma cells could induce significant antitumor responses and resulted in increased survival rates (56, 57). Menay et al. found that exosomes isolated from ascites of murine T cell lymphoma could markedly induce specific Th1 responses and increase CD4+ and CD8 + T cells by secreting IFN-γ, thus prolonging survival (101). Moreover, the efficacy of immunogenic activation induced by TEVs can be improved by incorporating stimulating drugs and TEV modification. For instance, Xiao et al. investigated antitumor efficacy after co-delivering DCs/pancreatic cancer cell-derived exosomes and cytotoxic drugs for treating pancreatic cancer. The authors found that including sunitinib and gemcitabine with DCs/Exo could significantly induce higher cytotoxic T-cell activity and more prolonged survival times than each treatment alone (102). As for TEV modification, Koyama et al. introduced a *Mycobacterium tuberculosis* antigen, early secretory antigenic target-6 (ESAT-6), into exosomes sourced from B16 melanoma cells through transfection and found that the engineered exosomes could remarkably suppress tumor growth in syngeneic B16 tumor-bearing mice. Dai et al. modified colorectal tumor-derived exosomes by incorporating IL-18 and indicated that these modified exosomes induced better DC maturation and T cell activation than unmodified exosomes. Similar results obtained by Yang et al. showed that engineered exosomes derived from IL-2-modifed ovalbumin (OVA)-expressing EL-4 lymphoma cells (Exo/IL-2) effectively inhibited tumor growth (103). These TEVs could trigger potent immune responses that may be beneficial in cancer immunotherapy. Notably, some TEVs also showed negative immunosuppressive properties and contributed to inhibiting immune activation and, thus, to evading immune surveillance. Multiple studies have recently focused on immune checkpoint receptor ligand PD-L1 that highly expressed TEVs and substantially contributed to immunosuppressive phenotypes in different cancer types (52–54). In this respect, the specific effects and functions of TEVs in immunity remain unclear, thus, more detailed studies associated with the therapeutic functions of TEVs are needed in cancer immunotherapy.

ICEVs, especially DCs-derived EVs, have been widely explored as the other primary source of EVs for cancer immunotherapy. Reports have suggested that these EVs carried MHC complexes and T cell co-stimulatory molecules that could activate T cells and present their antigens, thereby stimulating antigen-specific CD8+ and CD4+ immune responses for tumor suppression (86, 104). Moreover, DC-derived EVs have also been reported to activate natural killer (NK) cells and lead to the proliferation of NK cells for tumor suppression. According to multiple studies, trans-presentation of TNF, BAT3, NKG2D, IL-15Rα, and rhIL-15 in DCs-derived EVs were found to be the mechanism involved in NK cells’ ability to inhibit tumor growth (105–107). In clinical studies of EVs, DCs-derived EVs were firstly used to validate EVs’ safety and anti-tumor responses in cancer treatment. In 2005, DCs-derived exosomes pulsed with tumor antigenic peptides were found to be safe in patients with metastatic melanoma and non-small cell lung cancer in two phase I clinical trials (108, 109). Despite feasibility and safety of EV administration, the beneficial effects of the therapy seemed to be minor or negligible. In other phase II trials conducted in non-small cell carcinoma patients, exosomes derived from IFN-γ over-expressed DCs were reported to highly improve the function of NK cells; about 32% of patients experienced stabilization without tumor progression for over 4 months after chemotherapy. Although these clinical trials associated with DCs-derived EVs presented undesirable efficacy, the ICEVs’ therapy was found to be safe and feasible, paving the way for the ensuing clinical trials using EVs from other source or engineered EVs. Also, EVs that originated from T cells, NK cells, and macrophages can also be used as potent agents for inducing immunogenic functions in cancer immunotherapy. Growing evidence has indicated that regulatory T cell-derived EVs can work as suppressors against pathogenic Th1 responses through miRNAs (91). More specifically, Seo et al. reported that EVs collected from activated CD8+ T cells could be actively involved in interrupting fibroblastic stroma-mediated tumor progression by depleting tumor mesenchymal cells (**Figure 4B**). A similar activity was also observed in NK cell-derived EVs. Zhu et al. found that NK cell-derived EVs showed a significant cytolytic effect against multiple cells from glioblastoma, breast cancer, and thyroid cancer; the involvement of IL-15 could effectively increase the immunotherapeutic effects of NK cells-derived EVs (110). In addition to T cells and NK cells, EVs derived macrophages have also been considered a promising immunotherapeutic nanoplatform in combination with chemotherapy for treating cancer. Various chemotherapeutic drugs, such as PTX and DOX, have been encapsulated within macrophages-derived EVs. All these studies confirmed the therapeutic benefit of EVs, which significantly increased antitumor effects when combined with chemotherapeutic drugs (34, 72, 111, 112).

### Extracellular Vesicles Involved in Cancer Radiotherapy

Radiotherapy has been widely used to clinically treat localized cancer because of minimal invasiveness, low adverse effects, and outstanding selectivity and reproducibility. Rising evidence presented that EVs play a critical role in the cancer radiotherapy and greatly affect the tumor progression and therapeutic responses. As a primary light source, radiation has been well known as a powerful strategy to induce distinct tumor cell death process. During the apoptotic process, a series of pro-inflammatory cytokines including tumor-associated antigens (TAAs) and damage-associated molecular pattern (DAMPs) tend to be overexpressed, which can further trigger antitumor immunity. Based on this mechanism, several research found that radiation can also enrich EVs with a variety of TAAs and DAMPs and contribute to the antitumor immunity. In a study conducted by Lin et al., EVs derived from irradiated tumor cells were demonstrated to trigger antitumor immunity against tumor growth and metastasis by enhancing CD8+ and CD4+ T cells infiltration (113). Notably, a novel TAAs, CUB domain-containing protein 1 (CDCP1) was found in radiation-induced EVs and this antigen could be hopefully developed as a peptide vaccine for potent antitumor immune responses. In another study, Farias et al. showed that exosomes derived from MSCs, combined with radiotherapy, could lead to a significant enhancement of radiation-induced tumor apoptosis and metastatic tumor foci in a melanoma mouse model, suggesting that exosome-derived factors could be therapeutic after treatment of the tumors with radiation plus MSCs (114). These studies clearly presented a positive appearance of EVs induced by radiation against tumor; however, more reports indicated that radiation-derived EVs could exert negative effects on the cancer treatment. Growing evidence suggests that radiation-induced EVs are crucial driver in promoting tumor cell motility and assisting in the pre-metastatic niche formation in glioblastoma (GBM), lung cancer and head and neck squamous cell carcinoma (HNSCC). Arscott et al. reported that radiation-derived exosomes could significantly enhance the migration of receiving cells when compared to the exosomes derived from nonirradiated cells (92). These aggressively released EVs derived from cells after radiation exposure were altered in composition and became more oncogenic than normal EVs. According to the profiling results, radiation increased the exosomal connective tissue growth factor (CTGF) and insulin like growth factor binding protein 2 (IGFBP2) proteins, both of which were ascribed to favor tumor migration and progression (**Figure 4C**). Similarly, Zheng et al. recently found that tumor angiogenesis effect could be enhanced by exosomes when lung cancer cells were exposed to radiation. In this process, miR23 was enriched in these radiation-induced exosomes, subsequently mediating PTEN downregulation and facilitating tumor progression (115). Moreover, EVs has been widely identified as one of the crucial factors of in the development of radioresistance. Multiple studies found exosomes could increase radioresistance in GBM), lung cancer and HNSCC through exosomal miRNAs (e.g., miR-208a and miR-889), non-coding RNAs (e.g., circATP8B4 and AHIF), mRNA (e.g. CCND1, WWC1 and DNM2), and pathways crosstalk (e.g., AKT/mTOR and STAT1/NOTCH3) (116–120). Based on the aforementioned studies, EVs’ roles involved in phototherapy for cancer treatment are controversial and complicated, and could be affected by many factors such as various tumor types, tumor microenvironment and combinations with other therapies. As such, further preclinical and clinical studies are warranted for a more explicit version of EVs-based cancer phototherapy.

## Conclusion and Future Perspectives

Over the last decade, awareness in EVs in cancer development and progression has increased, and using EVs-based nanoplatforms for cancer treatment has advanced. We have discussed multiple kinds of EVs derived from different parent cells that are actively involved in tumorigenesis and progression, as well as their roles, functions, and potential in cancer diagnostics and therapeutics. Because of the specific roles of EVs in cancer development and promising potential application as therapeutic agents for cancer therapy, studies have focused on resolving limited production and inadequate content loading within EVs. Current engineering strategies, including both direct extracellular modification and indirect engineering of donor cells, make EVs as more attractive delivery vehicles in cancer therapy. In combination of conventional cancer therapies, such as chemotherapy and immunotherapy, the clinical use of EVs-based therapy will hopefully become feasible. Despite numerous exciting results in preclinical studies, many challenges remain and, thus, more systematic studies are needed to improve clinical translation. The main challenge EVs-based applications are facing is whether their use from allogenic sources will initiate immune responses or other negative responses. In other words, safety after uptake is a major challenge. As mentioned in earlier, EVs may function differently because they are delivery nanoplatforms derived from different parent cells sources. Some TEVs were found to have specific immunogenic responses for tumor suppression, while others may have immunosuppressive properties and contribute to inhibiting immune activation. Also, safety concerns exist. Therefore, more fundamental studies related to the specific roles of EVs from different sources in tumor development and their therapeutic functions are needed in EVs-based cancer therapy. Challenges in using engineered EVs and, especially, direct engineering strategies, include low EV recovery and limited cargos loading efficiency, which impair their application in cancer therapy to some extent. Several indirect intracellular manipulations that modified EVs through engineering parent cells have emerged, simultaneously increasing EV mass production and enriching cargos encapsulation. However, low isolation yield and inadequate purification of EVs after donor cell modification are current limitations along with potential safety issues. Moreover, because precisely quantifying active substances encapsulated in EVs is a concern, appropriate analytic platforms are required. Despite these challenges, EV-based cancer therapy is highly promising and may lead to advancing future cancer treatment.

## Author Contributions

All the authors contributed to the writing of the manuscript. All authors contributed to the article and approved the submitted version.

## Funding

This work was partially supported by the Innovation Program of Shenzhen (Grant No. JCYJ20180508165208399) (XL) and China Postdoctoral Science Foundation (2018M640834 and 2019T120756) (XL).

## Conflict of Interest

The authors declare that the research was conducted in the absence of any commercial or financial relationships that could be construed as a potential conflict of interest.
